# Can Non-Thermal Microwave Irradiation Inactivate Viruses? A Review with Some Computations

**DOI:** 10.3390/v18091022

**Published:** 2026-09-15

**Authors:** Gaspare Galati, Gabriele Pavan

**Affiliations:** 1 Department of Electronic Engineering (DIE), Tor Vergata University of Rome, via del Politecnico 1, 00133 Rome, Italy; gabriele.pavan@uniroma2.it; 2 Consorzio Nazionale Interuniversitario per le Telecomunicazioni (Italian Interuniversity Consortium for Telecommunications), Research Unit of Rome, via del Politecnico 1, 00133 Rome, Italy

**Keywords:** virus inactivation, microwave irradiation, non-thermal microwave, influenza, SARS-CoV-2, radiofrequency exposure

## Abstract

The use of non-thermal, human-safe electromagnetic (e.m.) waves in the GHz range to inactivate viruses has been proposed by a few groups. It is allegedly based on a transformation of a microwave photon into a phonon, which would produce a so-called Structure Resonance Energy Transfer (SRET) strong enough to break the capsid due to Confined Acoustic Vibration (CAV) at virus-specific frequencies. However, it is well known that e.m. photons’ absorption in water or in organic media does not generate single phonons at the same frequency. Rather, it generates many thermal phonons across a wide frequency spectrum according to the widely known microwave heating effect, as exploited, inter alia, in the microwave oven. However, there remains interest in proposed usages of non-thermal microwave fields to inactivate respiratory viruses for air sanitization purposes. Hence, this paper poses the question: can human-safe microwave irradiation inactivate viruses?

## 1. Introduction and Aim of the Paper

The contagion due to droplets and/or aerosol emitted by the normal human activities of breathing and speaking, as well as by coughing and sneezing, has been widely studied in the past [1,2,3,4,5,6,7,8,9,10,11,12,13], with a peak of interest during the COVID-19 period.

The standard strategies of virus disinfection use high temperatures, chemical agents and ultraviolet rays, with the latter generally in the UV-C1 band (λ=254 nm) [14,15,16,17,18,19].

In recent years, an alternative approach has been proposed by a few groups. It is based on electromagnetic fields, in particular, microwaves. The pertaining *leading papers* are by a group of researchers from Taiwan University [20,21,22]. Despite the hypothesis shown in [20,21,22] and in some ensuing papers analyzed in the following text, how non-thermal microwaves affect virus infectivity is an unresolved problem today.

Hence, we intend to comment on the alleged inactivation of respiratory viruses by human-safe, non-thermal microwave irradiation. The analysis in [23] confirms some general problems in the worldwide research assessment and presents proposals aimed at fixing these problems in Sections 4–6 of [23], to which the interested reader is directed.

From a general point of view, this paper aims to stress the need for correcting actions with regard to misleading publications, as tested by the ERROR (Estimating the Reliability & Robustness Of Research) project launched in 2024 by the University of Bern [24]. In fact, misleading and fabricated publications are increasingly produced [25], with rapid broadcasting on the Internet and spreading also into the most critical area, the medical literature.

This work is organized as follows. Section 2 addresses the biophysical context. Section 3 analyzes—and criticizes, when needed—the pertaining publications. Section 4 includes a quantitative examination of the literature on virus inactivation by microwave radiation. Section 5 presents and analyzes a proposal for electromagnetic modeling and for the evaluation of the field intensity level needed. Comments and conclusions are presented in Section 6.

In this work, we analyze the direct interactions of human-safe microwaves with respiratory viruses, not considering in detail any indirect microwave-related mechanism, such as dielectric heating, localized heating, and absorption by the surrounding medium or by microwave-responsive materials.

## 2. The Threat of Respiratory Viruses

**Figure 1:**
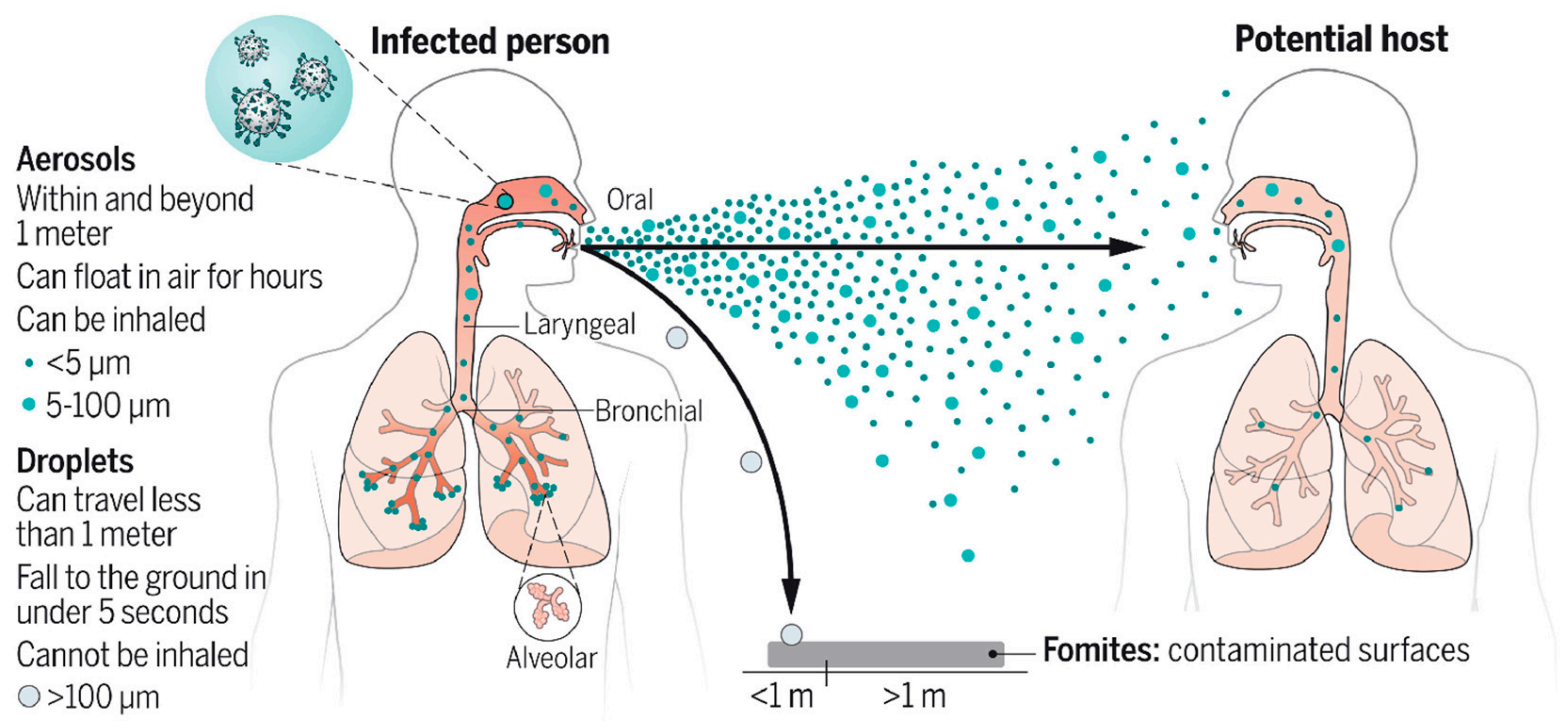
Airborne transmission of respiratory viruses, from [6] (Creative Commons Attribution 4.0 International, CC BY 4.0 license).

Humans produce respiratory droplets ranging from about 0.1 μm to about 1 mm [3]. Smaller aerosols (≤5 μm) are buoyant in air and thus can be affected by air flow, which can move them over metric distances. On the other hand, droplets with a diameter >100 μm quickly fall (Figure 1, CC BY 4.0 license).

Aerosols and droplets produced by an infected individual may contain infectious viruses (Figure 1).

The most dangerous interval for the infected particles is between 0.1 μm (100 nm, the typical size of a respiratory virion) and 5 μm, sometimes extended to larger droplets up to 100 μm in diameter. Estimates based on average SARS-CoV-2 viral loads *in sputum* indicate that one minute of loud conversation could generate over 1000 aerosols containing virions. Assuming the high viral titers for infected super-emitters (with a viral load 100 times higher than the average), this results in over 100,000 virions in droplets emitted per minute of conversation [3].

A diameter of 5 μm represents the limit for the largest particle size that can remain suspended in still air for more than 5 s from a height of 1.5 m. These particles reach 1 m to 2 m from the emitter (typically depending on the velocity of the airflow) and can be inhaled.

The size diameter distribution of respiratory aerosols, as reported in [6], is multimodal, with peaks around 0.1 μm, 0.2−0.8 μm, 1.5−1.8 μm, and 3.5−5.0 μm, each one from a different generation site, production process, and expiratory activity. The smaller the size, the deeper the aerosols originate in the respiratory tract. A larger mode centered at 145 μm for talking and 123 μm for coughing originates mainly from the oral cavity and lips. Most exhaled aerosols are <5 μm, with a large fraction <1 μm, for most respiratory activities, including those produced during breathing, talking, and coughing. Normal breathing has been shown to release up to 7200 aerosol particles per liter of exhaled air [6]. An analysis including measurements and simulations is presented in [26]; this study reports the size distribution of droplets emitted by an infected individual along with their corresponding emission rates but also provides a detailed simulation of their dispersion in indoor environments through both Eulerian and Lagrangian approaches. In particular, it shows that particles with diameters smaller than 1 μm exhibit significantly higher emission rates (approximately 217.6 particles/s), thus playing a dominant role in airborne transmission. These values are derived from the works [27,28].

In samples collected from influenza patients while breathing, talking, and/or coughing, more than half of the viral RNA was found in aerosols <4 μm to 5 μm. In still air, a 5 μm aerosol takes 33 min to settle to the ground from a height of 1.5 m, whereas a 1 μm aerosol can remain suspended in air for a time greater than 12 h [6].

In [7] it is shown that in quiescent ambient air, respiratory particles of 5 μm take approximately 30 min to fall to the ground from a height of 1.5 m; a 10 μm respiratory particle takes 10 min to fall from a height of 2 m and a 1 μm particle takes 16 h to fall the same height. This timescale leaves ample time for transport and inhalation exposure at long distance. The related infection problems are discussed in [8,9,10,11,12,13]. In [29], the stability of SARS-CoV-2 and SARS-CoV-1 in aerosols and on various surfaces is evaluated and decay rates are estimated.

In [10], it is shown that droplets/aerosol in long-range airborne transmission generally support a one-virion assumption for most viral load ranges. However, for short-range airborne transmission, the impact of multi-virion aerosols on infection risk must be considered. Finally, ref. [12] points out the predominance of small particles (<5 μm) within infectious aerosols.

Table 1 shows the main parameters for the Influenza A and SARS-CoV-2 viruses [30,31].

To define a sanitization process capable of destroying viruses, the European Regulation (EN 14476) fixed an inactivation threshold in viral titer of at least 4−log. This means that on $10^{4}$ initial viral particles, a maximum of one particle survives, i.e., an inactivation rate of 99.99% is reported.

## 3. Proposals to Inactivate Viruses by Electromagnetic Irradiation

### 3.1. Thermal/Non-Thermal Effects for Deactivation of Viruses

In principle, electromagnetic waves can produce thermal or (by some alleged physical resonance) non-thermal effects on viruses.

Thermal effects increase the surrounding temperature, inducing drastic changes in the virus morphologies that are capable of inactivating them. In [32], the potential use of electromagnetic radiation is analyzed for thermal inactivation of pathogens within aerosolized droplets on the micrometer-length scale. For saline solutions, the authors of [32] found that inactivation is significantly enhanced for frequencies in the range 10–100 GHz, due to the increase of the saline solution’s electrical conductivity over this frequency range.

The preliminary study in [33] analyzes the possibility of using heating (due to the strong absorption of the radiation around 2.15 GHz) to destroy SARS-CoV-2 and related viruses, like the operation of a microwave oven. A thermal disinfection at 60 °C for 30 min, 65 °C for 15 min and 80 °C for 1 min is effective in strongly reducing SARS-CoV-2 (coronavirus) infectivity by at least 4−log. The nucleo-capsid protein of SARS-CoV-2 is completely denatured in 10 min at 55 °C. All results in [33] are obtained with coronaviruses in suspension.

The Ph.D. thesis. in [19] analyzes four mechanisms to inactivate the SARS-CoV-2 coronavirus: High temperature, Ultraviolet-C radiation, Chemical disinfection, and finally “Inactivation of pathogens using electric fields on a microchip platform”. The latter method considers DC or kHz fields, not microwave; no mention of microwave, Structure Resonance Energy Transfer (SRET) or Confined Acoustic Vibrations (CAVs) is present in this 206-page Ph.D. thesis, defended publicly on 24 April 2023 at the Delft University of Technology.

The deactivation of viruses based on non-thermal effects is analyzed and reviewed in [34], where important recommendations are reported regarding the research directions for the future, as follows.

(a) *“The development of an effective and specific method/equipment that can detect the nonthermal effects of MWs can be repeated by anyone. The debate about the nonthermal effects is due to the lack of an effective method/equipment that can detect the nonthermal effects of MWs directly”.*
(b) *“Experiments should be repeated in different laboratories with the same function. This kind of academic activity can ensure the effectiveness of the discussion of the existence of non-thermal effects based on experimental phenomena”.*
(c) *“The nonthermal effects should be explained from the microcosmic angle, namely, in terms of molecules, atoms, and electrons. Each researcher in a related study field should try to explain the essence, not only report his or her experimental findings, even if the statement about the essence differs from the statements of other researchers”.*
(d) *“A universal mechanism should be extracted. The proposed mechanisms of the nonthermal effects of MWs differ from one another because researchers have different research backgrounds and knowledge structures. These mechanisms cannot explain all or most experimental phenomena. Thus, it is necessary to establish a universal mechanism”.*

### 3.2. The Literature on the Inactivation of Viruses: 2009–2015

To describe the mechanism of absorption of far infrared radiation by solid nanoparticles of titanium dioxide ($TiO_{2}$), the authors of [35] considered a model in which the electrons move outwards, leading to a shell of negative charge surrounding the nanoparticle, with its interior slightly positive. A simplifying assumption is that the nanoparticle has an outer shell of negative charge, with total charge equal to q, while the interior of the nanoparticle is positively charged, so that the nanoparticle is neutral overall. Hence, to describe the power absorption of a nanoparticle due to the interaction with an external e.m. field, the authors used a simple damped mass-spring model (see Appendix B). In this case (solid nanoparticles of $TiO_{2}$, i.e., a crystalline solid matter in a highly ordered, periodical three-dimensional lattice structure), photon-to-phonon conversion is possible. On the other hand, in irregular organic structures this conversion is limited to the random generation, by each e.m. photon, of many thermal phonons, as in the well-known microwave oven.

In [36], modeling viruses as spherical particles immersed in a medium, low-frequency vibrational modes were calculated using the elastic continuum theory. In 2008, a group of researchers from National Taiwan University described a resonance-enhanced dipolar interaction between terahertz photons and confined acoustic phonons in nanocrystals [37]. From the measured transmission spectra, they observed that the photon frequency of the resonant absorption is inversely proportional to the diameter D of the nanocrystals and agrees with that of alleged dipolar active *confined acoustic vibration* (CAV) modes. Considering the elastic properties of viruses [38], they derived the alleged resonance frequency as $f=V_{L}/2D$, where $V_{L}$ is the longitudinal sound velocity and D is the diameter of the particle. This concept was taken up again and applied to viruses [39,40]. For Influenza A (Table 1), with D~100 nm and $V_{L}=2400\;m/s$, the derived resonance frequency is about 12 GHz.

According to some proposals to inactivate viruses by electromagnetic irradiation [20,21,22], virions resonate (in a so-called confined acoustic dipolar mode) with electromagnetic waves of the same frequency, thus leading to an alleged *structure resonance energy transfer* (SRET), that is, a transformation of electromagnetic waves into *confined acoustic vibrations* (CAVs), inducing the fracture of the virus structure.

The assumptions of SRET and CAVs come, as said, from *forerunner papers* [20,21,22]. The proposal of a *microwave resonant absorption* (MRA) involving CAVs is first found in [20] (2009), where microwave absorption peaks in virions are attributed to dipolar coupling, causing CAVs of viruses to modify the dipole moments and resulting in some microwave resonant absorption (MRA). For example, for Enterovirus 71 (diameter ~30 nm), a resonant absorption is alleged at 45 GHz; for Influenza A virus (diameter ~100 nm), it is around 12 GHz, with a higher order mode at 26 GHz.

In [21], it is shown that the hydration levels on the capsid surface of viruses can affect the bandwidth of the MRA induced by CAVs. A viscous medium could strongly dampen the vibration and decrease the quality factor of dipolar modes; see also Appendix B.

From a mechanical point of view, the SRET mechanism was mathematically analyzed in [26].

#### Some Comments

The analysis proposed in [22] (by National Taiwan University, as [20,21,37]) is based upon the undemonstrated hypothesis that a spherical virus is *“like a homogeneous sphere but with opposite and equal charges in the core and shell regions”* (page 3 of [22]). Another not-scientific claim is shown in the introduction of [22]: *“Theoretically this SRET process is an efficient way to excite the vibrational mode of the whole virus structure due to a 100% energy conversion of a photon into a phonon of the same frequency”*. Following this approach, a mechanical model (the damped mass-spring model, Appendix B) is used, or more accurately, misused, to estimate the microwave power threshold for virus inactivation.

In [22], the microwave absorption spectrum of Influenza A H3N2 viruses (diameter ~100 nm) showed a resonance peak at 8.2 GHz (see Section 5.1). A low inactivation level (38%) of H3N2 viruses was observed when illuminating the viral solution by $82\;W/m^{2}$, using an 8 GHz microwave. A 100% inactivation level was obtained at a power density roughly 6.7 times higher than the IEEE Microwave Safety Standard value for people in open public spaces [41] which, for frequencies in the range 3–96 GHz, is given by the formula: $100{\left[\frac{f(GHz)}{3}\right]}^{1/5}\;W/m^{2}$. At 8.2 GHz, this limit corresponds to as much as $122\;W/m^{2}$ (214 V/m).

Note that most national regulations establish much lower limits than IEEE; in Germany and France, the limit is $4.5\;W/m^{2}$ (41.2 V/m); in Poland, Switzerland and Italy, the limit is $0.095\;W/m^{2}$ (6 V/m), a value recently extended (Italian Law No. 214/2023 for compatibility with the 5G system) to 15 V/m, i.e., about $0.6\;W/m^{2}$. Therefore, the value of $122\;W/m^{2}$ in [22] is 203 times higher than specified in the Italian regulations.

### 3.3. The Literature on the Inactivation of Viruses: 2015–2025

The authors of a recent paper [42], using an adapted co-planar waveguide (CPW), studied which frequencies could potentially neutralize SARS-CoV-2 virus-like particles. They showed that treatments with an exposure time from two to ten minutes at frequencies between 2.5 and 3.5 GHz and with an electric field as large as 413 V/m reduced infectivity but did not alter the main structures of coronaviruses, i.e., the Spike, Nucleocapsid, Envelope and Membrane proteins.

They concluded that *“These findings do not support a physical resonance model that destroys virus particles and/or viral proteins. However, we cannot entirely rule out the possibility that the frequencies used in our study did not physically rupture virus particles. Future studies should seek to address this using electron microscopy”*.

At the inception of COVID-19 (early 2020), the Air Force Research Laboratory (AFRL) scientists started to investigate the potential usage of electromagnetic radiation to sterilize areas contaminated with the SARS-CoV-2 virus, with the main aim to protect military medical personnel.

The starting point of this research was to assess the ability of electromagnetic radiation to reduce the infecting capability of airborne coronavirus. The AFRL project, named “Viral Inactivation by Directed Energy Radiation (VIDER)”, began on 20 March 2020 (https://www.afmc.af.mil/News/Article-Display/Article/2163064/afrl-scientists-investigate-can-microwaves-reduce-viability-of-airborne-coronav/, accessed on 15 June 2026).

Starting from the fact that the Spike (S) protein on the surface of the viral membrane is responsible for the viral entry into host cells, the methods to inactivate the entry of SARS-CoV-2 through disruption of the S protein binding to its cognate receptor on the host cell were analyzed in [43] and [44], from the same research group at the Air Force Research Laboratory in USA. Using a Molecular Dynamics (MD) simulator, ref. [43] explores the effects of electric field orientations on the spike proteins of SARS-CoV-2 virions. In [44], the dynamic motion of the SARS-CoV-2 spike protein was simulated, and a resonance frequency at 7.3–7.4 GHz for the intrinsic vibration of the spike protein (which is different from the previously proposed viral shell-core dipole model) was identified, with the authors reiterating that *“measuring natural vibrational frequencies of a single virion in a biological environment is challenging. Assigning structural features to measured spectra is even more difficult”*.

#### Some Comments

From the above analysis, we underline that the recommendations proposed in [34] (see Section 3.1) are not respected, and different experimental and simulation studies on measuring the natural vibrational frequencies for SARS-CoV-2 have estimated different values.

Moreover, some recent articles show a strong emphasis on the use of microwaves to deactivate viruses. This is the case of [45], which deserves some analysis.

First, its authors declared that, after an automated search providing 305 papers, only 16 were considered; of course, such a low number makes the review in [45] of little interest. Note that a more extensive review of twenty-two publications before 2022 is available in [46], a paper which is not discussed at all in [45].

Second, ref. [45] has an *advertising style,* with as many as thirteen very similar sentences (i.e., *the microwave technology … within the GHz range … adhering to strict regulatory standards … has emerged as a … method for mitigating the airborne transmission of … respiratory viruses including SARS-CoV-2…*) declaring an alleged inactivation of respiratory viruses by microwave. Nothing is written in [45] about its (uncommented upon) Reference No. 11, i.e., the paper [47] by Cantu et al., which mentions a 77% inactivation effectiveness only, versus the much larger values requested for clinically significant biocides, typically 99.99%.

Finally, in the Summary and Introduction of [45], we read, *“Non thermal microwave (MW) irradiation … through selective resonance energy transfer (SRET) … disrupts viral structures through vibrational resonance mechanisms … to strengthen infection prevention …. Microwave resonant absorption (MRA) … arises when MW frequencies align with the Confined acoustic vibration (CAV) modes of spherical or rod-shaped virions … this technology operates at MW power density significantly below … 100 W/m^2^”*. On the other hand, in Table 1 of [48], the Influenza A virus H3N2 in solution was irradiated in a frequency range of 6 GHz–12 GHz; a 100% inactivation ratio was achieved at an alleged resonance frequency of 8.4 GHz with a power density as large as $810\;W/m^{2}$.

In Section 3 of [49], we read, “Test were performed by UNIMI (University of Milan) staff” and “Tests have been conducted by UNIMI staff with the supervision of ViroStatics operator”. However, these two organizations are not specified as authors of the papers, whose authorship is instead attributed to personnel from the Elettronica Co. Moreover: “*The viruses were exposed to the following conditions: frequency from 8 to 11 GHz, electromagnetic field amplitude close to 6 V/m for a duration of 600 s. The results (Tables I and II in Section III C) show an inactivation of 87% for SARS-CoV-2 and >90% for H1N1 viruses. Aerosol particles were generated with size of 1 μm”*. These data are commented on together with the content of the ensuing paper [50], written by the same group.

The following claims in [50] are not confirmed by results: “*Through an experimental in vitro model, the resonant energy transfer effect from microwaves to the virus was demonstrated, effectively inactivating the Wuhan variant of SARSCoV-2 in aerosols. Importantly, the microwave power density used in this technique remains well within the safety limits set by worldwide regulatory agencies*”. In the experiment described in [50], particles with a size up to 10 μm were generated with a commercial aerosol generator by Omron. Commercial generators optimize the particle’s size for their absorption in the human respiratory system, and their drop size distribution is mostly concentrated on the 5–10 micrometer interval [51]. This is strongly different from the interval of aerosols generated by breathing (and speaking) [52,53], which start at about 0.3 micrometers, and it is well-known that tidal breathing generates particles mainly sized between 0.3 and 0.5 micrometers. This discrepancy invalidates the results of [50] and, from the same authors, [49].

Another important aspect is the power level. In [54] we read, “*MS2 coliphage used as a human model virus was aerosolized and exposed to the direct microwave irradiation for ~2 min at three different power levels (700, 385, and 119 W). … Direct exposure of airborne MS2 viruses to the microwave irradiation at 700 W for less than 2 min was shown to result in more than 90% inactivation efficiency, about 65% at medium power level (385 W), and 50% at the lowest level (119 W). The aerosol inactivation rate followed a linear relationship with the microwave exposure time (R^2^ = 0.9889)*”.

Moreover, in both papers [49,50] the analysis neglects the radiated power level, specifying only “*about 6 V/m*”, with no data on the antenna pattern and the input power. The e.m. field in the aerosol chamber is expected not to be uniform. This important feature is shown in the color-graph of Figure 1 of [55], another paper by this group, where the average value in the chamber is of the order of 120 to 140 V/m, with a peak value of 200 V/m and a minimum of 40 V/m. This means that the power levels in [55] are an order of 400 or 500 times those in [49,50], creating a large discrepancy.

There are two aspects of the results in [55] that do not appear compatible with the alleged SRET/CAV.

(i) The wide-band frequency response; see Table 1 of [55] showing the percentage reduction in A (H5N1) viral titers across different microwave frequency bands. The related results contrast with resonance, which is intrinsically a narrow-band phenomenon.

(ii) The strong dependence on the exposure time of the percentage of inactivation, measured by the H5N1 viral titer reduction; see Table 2 of [55], which contrasts with SRET/CAV being an allegedly near-instantaneous phenomenon.

In the discussion of [55], the authors honestly admit that “*the methodology employed in the present study differs from that of Banting* et al. *[Electromagnetic deactivation spectroscopy of human coronavirus 229E (2023). Sci. Rep. 13, 8886.], who developed a system specifically optimized for precise control over electromagnetic field distribution using custom-designed microwave guide sections*.” and that “*The system’s complexity, coupled with the lack of measurable data during the aerosolization and collection phases, hindered a quantitative analysis …”* and finally, that *“…the SRET methodology primarily relies on field amplitude and is not directly dependent on dosimetric evaluations”*.

A minor discrepancy is found in the final declaration of competing interests in [55], where we read, “The remaining authors declare that they have no conflicts of interest related to this work”; they are Silvio Brusaferro, Gaetano P. Privitera and Alberto Sangiovanni Vincentelli. While in the Conflicts of Interest Statement in [50], we read “*A. Sangiovanni-Vincentelli receives consulting fees from Elettronica S.p.A., sponsoring the study*”.

Contrasting results are presented in [47,56,57,58]. In [47], we read: *“From this study, we cannot prove that SRET is occurring …. However, we did find that the overall effect appeared to be much less defined than in previous publications. This would suggest that another mechanism can account for the loss of viral infectivity during RF exposure” and “Therefore, at this time we cannot recommend the use of RF technologies to neutralize coronavirus …”*.

Summing up, in Appendix A it is shown that an e.m. wave of centimeter length presents a very weak interaction with micrometer (or nanometer) objects such as aerosol particles and viruses.

## 4. Quantitative Examination of the Literature on the Inactivation of Viruses Using Microwaves

Using Scopus, one of the largest multidisciplinary bibliographic and citation databases of the scientific literature (https://www.scopus.com/pages/home#basic, accessed on 4 June 2026), we implemented a paper search (time span: 2008–2025) based on the words viruses inactivation, microwaves resonant absorption, Confined Acoustic Vibrations, Structure Resonance Energy Transfer (with the acronyms CAV and SRET) in the title or in the abstract or in the keywords.

**Figure 2:**
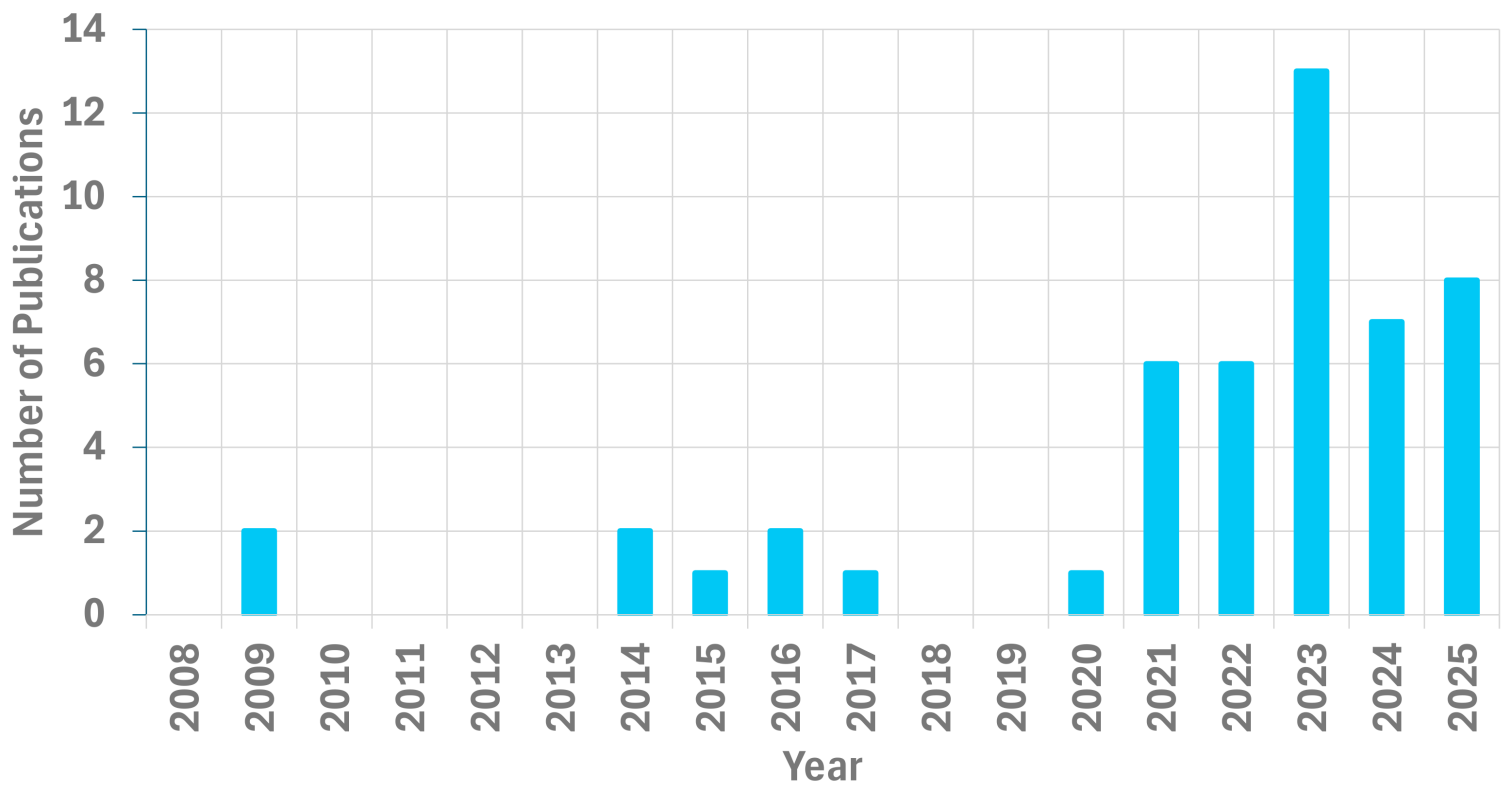
Articles related to virus inactivation by radiated microwaves, by year from 2008 to 2025.

After manual refinement, eliminating a low-quality paper published in a predatory journal and adding other relevant documents found in the References of the previous papers, 49 documents were selected. They include references [20,21,22,26,40,42,43,44,45,46,47,48,49,50,54,55,56,57,58,,59,60,61,62,63,64,65,66,67,68,69,70,71,72,73,74,75,76,77,78,79,80,81,82,83,84,,85,86]. Figure 2 shows the number of publications by year.

The publications are distributed as follows: Journal 81.6%, Conference Proceeding 14.3% and, finally, one Report and one Patent 4.1%. The year 2023 shows the maximum interest, obviously related to the COVID-19 pandemic in the years 2020–2022.

The distribution by country is led by the United States, Italy and Taiwan.

**Figure 3:**
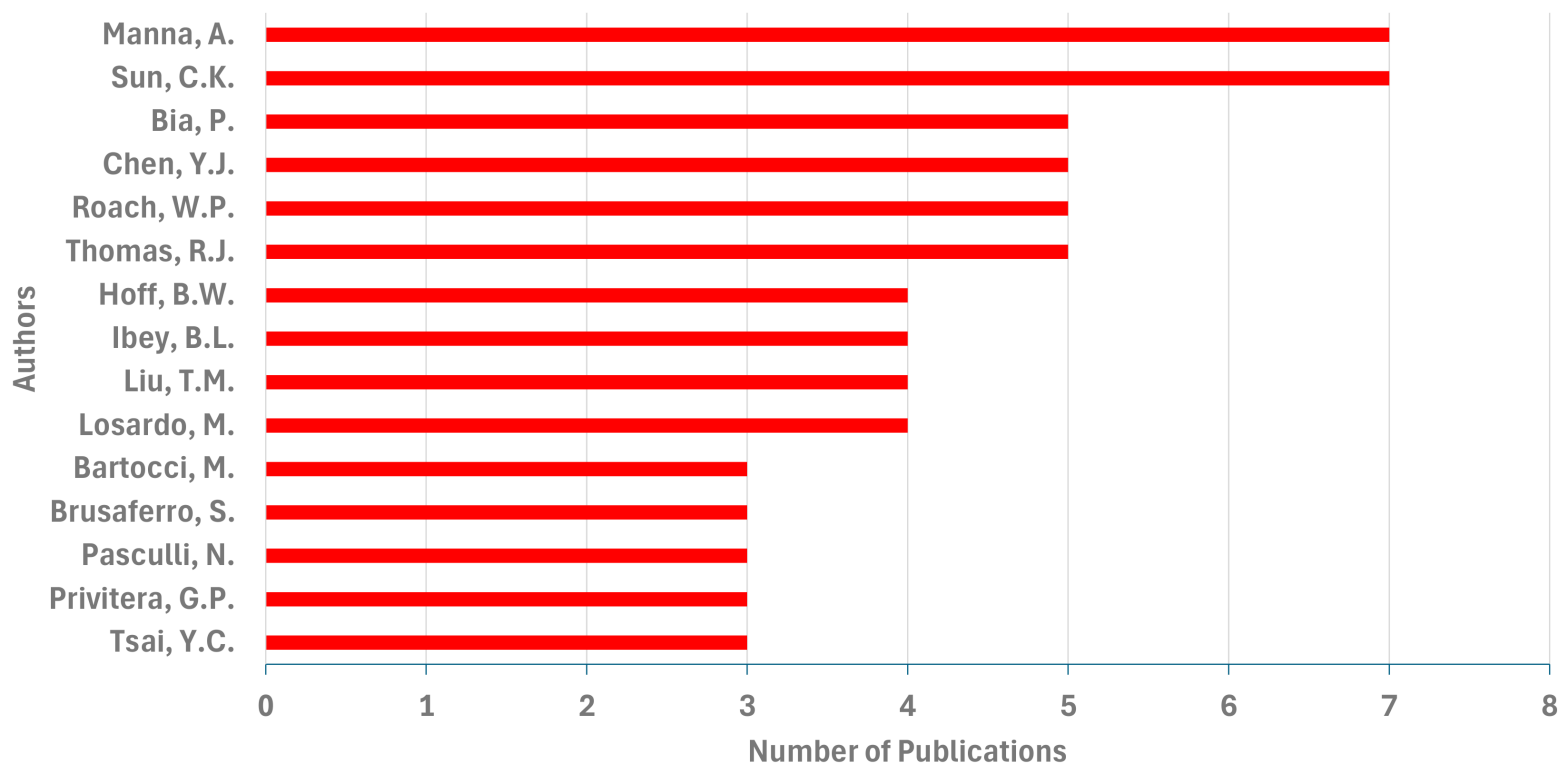
Articles (2008–2025) on virus inactivation by radiated microwaves, by author.

Figure 3 shows the number of articles by authors with at least three publications. The most prolific authors are Sun C. K., Chen Y. J. and Tsai Y. C. (from the Department of Electrical Engineering and Graduate Institute of Photonics and Optoelectronics, National Taiwan University, Taipei, Taiwan), and Manna A., Bia P. and Losardo M. (from Elettronica S.p.A, Rome, Italy).

In 2026, two papers [87,88] have been published. In [87], the alleged inactivation ability of SRET/CAV is proposed once again, by the Elettronica SpA group, as an efficacy sanitization technology against enveloped viruses, including the human respiratory syncytial virus. Tests are executed at 21 °C, while the infecting aerosols/droplets are, of course, emitted at about 36 °C. In Figure 4 of [87], a colorbar shows e.m. field intensity values between 500 V/m and 40 V/m, much higher than the “human-safe” ones.

In [88], investigations led by the Air Force Research Laboratory (USA) are presented involving the interaction between 4.0 GHz microwave radiation and bovine coronavirus (BCoV), with the aim to better elucidate the roles of thermal and non-thermal effects in radio frequency (RF)-induced viral inactivation. Despite viruses being exposed to high-RF electric field amplitudes (41.5±5.2 kV/m), analysis via TCID_50_ assays revealed no statistically significant reduction in virus survival as compared to controls, agreeing with prior observations that higher frequencies (~7–8 GHz) produce more pronounced— although limited—inactivation effects, while lower frequencies show a reduced efficacy, hence highlighting the need for further studies.

## 5. On the Proposals for Electromagnetic Modeling of Virions in View of Their Inactivation

### 5.1. Modeling the Electromagnetic Interaction

In [22], it is hypothesized that viruses, like Influenza A, can be modelled as a spherical structure that resonates at microwave frequencies (order of a few GHz). Such a resonant absorption mechanism is assumed through dipolar coupling with some alleged confined acoustic vibrations.

This modeling treats virions as spherical homogeneous nanoparticles whose resonance absorption frequencies are those predicted by the elastic continuum theory.

**Figure 4:**
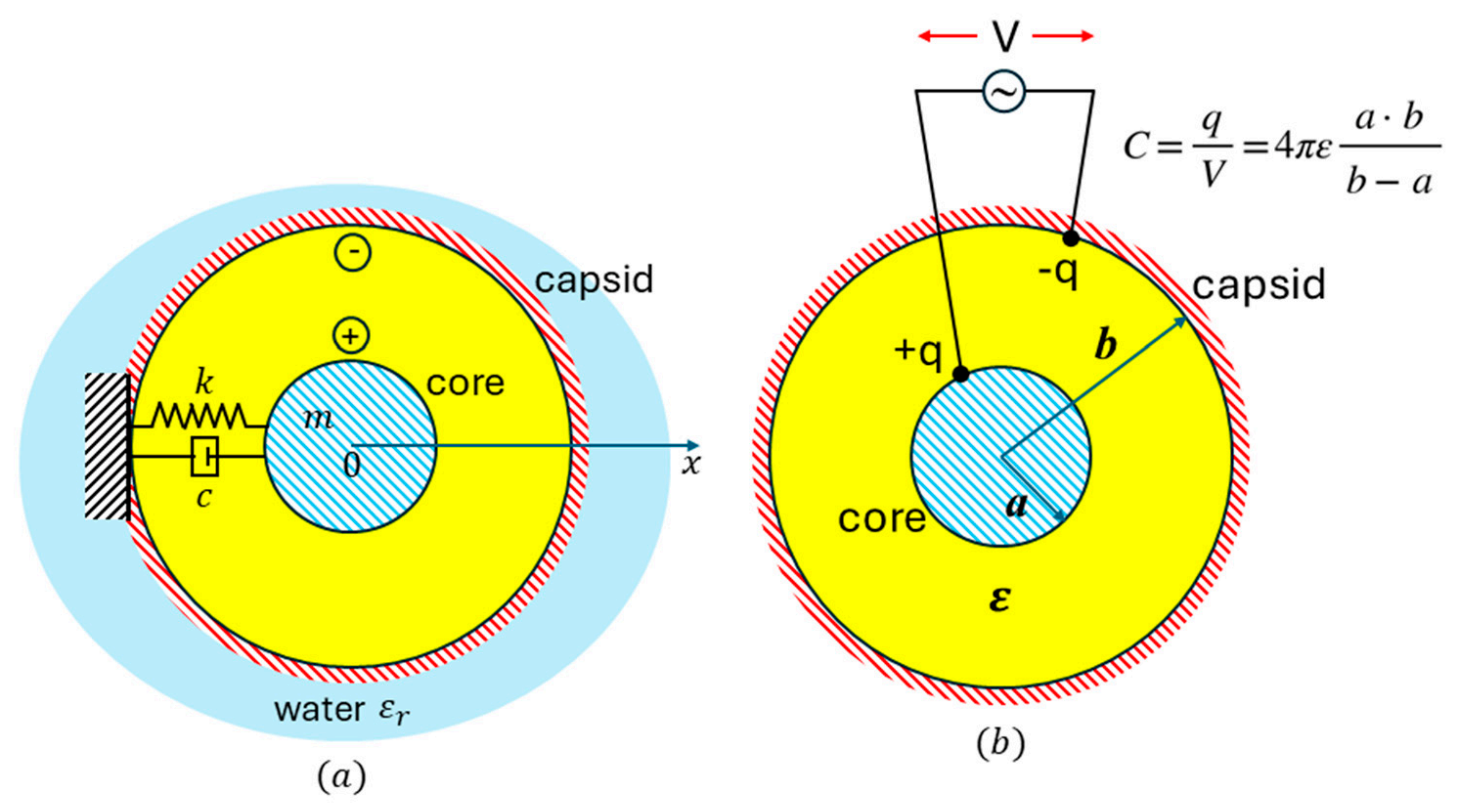
(**a**) A dynamic model of a virion in liquid water with relative permittivity $\varepsilon_{r}$. (**b**) The simplified model (used in [22]) of a dipole with an external shell (capsid, negatively charged) and an internal core (positively charged).

Mixing some electrical concepts with mechanics ones, a *damped mass-spring system*, with a mass m, a stiffness of the spring k and a damping coefficient c, is used to model the virions (see Figure 4a), ignoring the water or the water-like material (droplets, aerosols) around the virions.

In Figure 4b, the model is simplified as a dipole with an external shell (capsid, negatively charged) and an internal core (positively charged), forming a spherical capacity $C=\frac{q}{V}=4\pi \varepsilon \frac{a\cdot b}{b-a}$, where ε is the dielectric permittivity between the core and the capsid.

In a damped mass-spring system (as described in Appendix B), the movement along direction x due to an external stimulus, i.e., the force $F_{a}(t)$, is regulated by the well-known differential equation:

(1) $$m\frac{d^{2}x\left(t\right)}{dt^{2}}+c\frac{dx\left(t\right)}{dt}+k\cdot x\left(t\right)=F_{a}\left(t\right)$$

**Figure 5:**
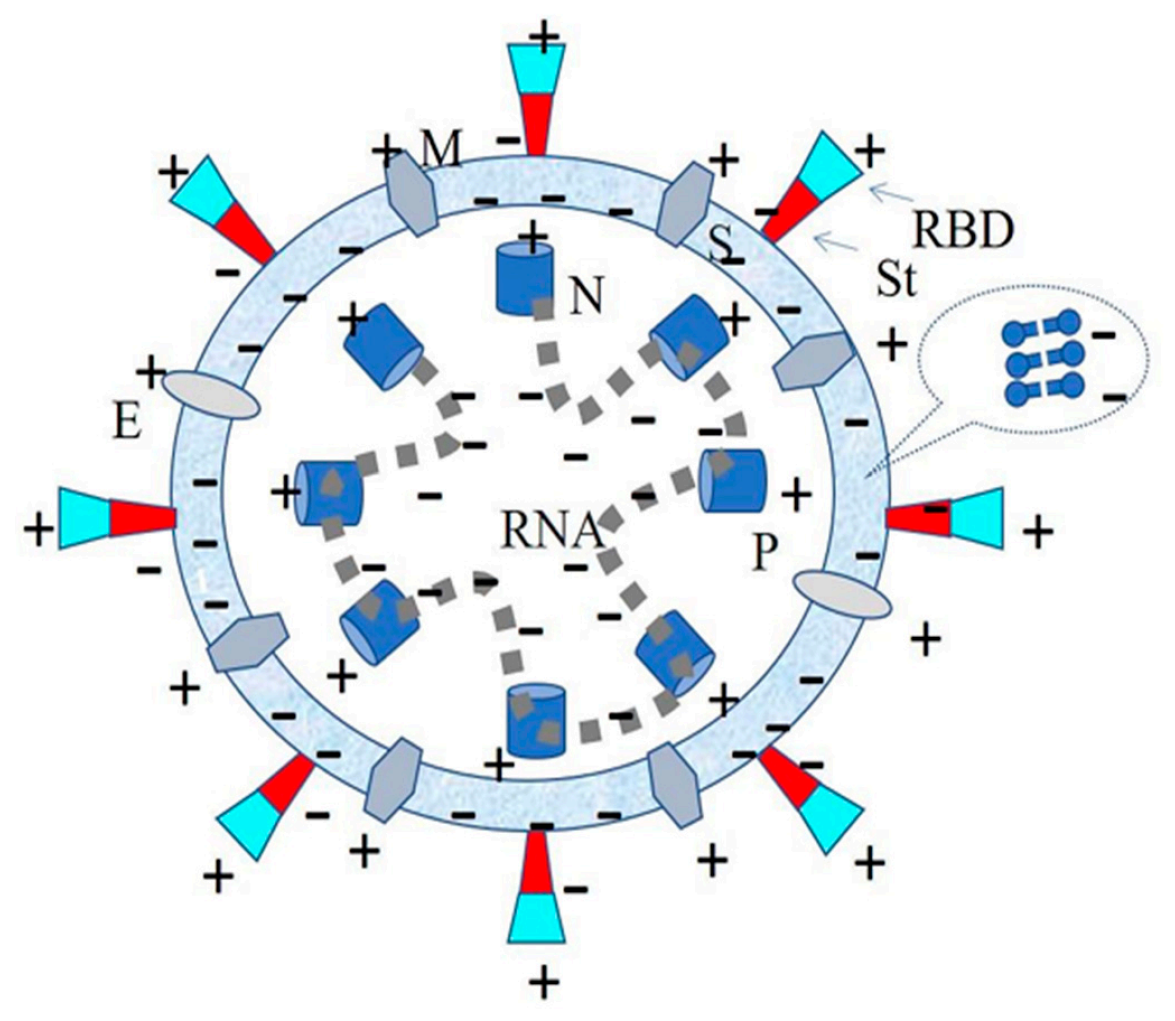
Spherical approximation of a SARS-CoV-2 virus (secondary and tertiary structures are neglected) with the charge distribution (from [69], Creative Commons Attribution 4.0 International, CC BY 4.0 license).

With the harmonic stimulus generated by an electric field $E\left(t\right)=E_{0}cos(\omega t)$, the force is $F_{a}\left(t\right)=q\cdot E(t)$, where q is the charge on the virion and $E_{0}$ is the incident electric field strength. The solution of Equation (1) results in (see Equation (A16) in Appendix B):

(2) $$x\left(t\right)=qE_{0}A\left(\omega \right)cos\left\{\omega t+\theta \left(\omega \right)\right\}$$

where $A\left(\omega \right)$ and $\theta \left(\omega \right)$ are the amplitude and the phase of the frequency response of the system (see Appendix B), normally expressed in terms of three parameters: the undamped resonance frequency $\omega_{0}=\sqrt{\frac{k}{m}\;}$, the damping ratio $\zeta =\frac{c}{2\sqrt{k\cdot m}}=\frac{c}{2m\omega_{0}}$ and the quality factor of the resonator $Q=\frac{1}{2\zeta }$. Of course, this is an oversimplified model: in [69], a much more realistic (and complicated) model for the charge distribution of a SARS-CoV-2 virion is described as shown in Figure 5 (from [69], CC BY 4.0 license).

In this model, the charge distribution is uneven and is related to the main components of the virus, i.e., RNA, nucleocapsid proteins (N), membrane phospholipids (P), membrane proteins (E and M), and surface spike proteins (S), with the characteristic receptor-binding domain (RBD) and stalk (St), mostly covering the main functional subunits of the spike protein, S1 and S2.

Using such a model, for the SARS-CoV-2 virus, the total charge of the virus is estimated to equal $4650\;e^{-}$ [69], and many resonant frequencies (see also Appendix B.2) result.

### 5.2. Overall Evaluation of the Electric Field $E_{0}$ Able to Inactivate Viruses by an Alleged Electromagnetic-to-Acoustic Resonance Energy Transfer

With the simplified model of a virus as a damped mass-spring system (Figure 4), an attempt to estimate the threshold of the electric field $E_{0}$ able to inactivate viruses is described in [22], where the induced stress at the resonance frequency $\omega_{0}$, i.e., $P_{stress}(\omega_{0})$, is assumed to be at least twice the maximum value of the force, i.e., $2\cdot x_{max}\cdot k$ (that from Equation (2) is equal to $2\cdot \left\{qE_{0}A\left(\omega_{0}\right)\right\}\cdot k$) over the 58% of the shell region in the equatorial plane of the spherical virus, i.e., $0.58\cdot \pi r^{2}$, where r is the radius of the virus. The formula is as follows:

(3) $$P_{stress}\left(\omega_{0}\right)=\frac{2qE_{0}A\left(\omega_{0}\right)k}{0.58\cdot \pi r^{2}}=\frac{2qE_{0}Q}{0.58\cdot \pi r^{2}}$$

where q is the charge, Q is the quality factor of the resonant model, $E_{0}$ is the incident electric field strength and $A\left(\omega_{0}\right)$ is the amplitude of the frequency response of the system at the resonance frequency. The second equality comes from $A\left(\omega_{0}\right)=\frac{Q}{k}$ (see Equation (A13) in Appendix B).

Inverting Equation (3), the alleged threshold ($E_{0}^{T}$) of the electric field results in

(4) $$E_{0}^{T}=0.58\cdot \pi r^{2}\frac{P_{stress}\left(\omega_{0}\right)}{2qQ}$$

where r, $P_{stress}$ and the charge q depend on the particular virus.

With r and $P_{stress}$ taken from the literature, ref. [22] shows an attempt to estimate the charge q and the other parameters such as $\omega_{0}$ and Q by some experimental data of the microwave absorption spectrum of viruses. In general, an absorption experiment requires an electromagnetic source (over the frequency range 1–40 GHz) and an electromagnetic detector. In [22], a sample of viruses is posted between the source and the detector; in the presence of the sample, the detector records the measured power, $P_{s}\left(\omega \right)$. Then, the power is measured without the sample, $P_{0}\left(\omega \right)$, for control purposes.

The ratio $\frac{P_{s}\left(\omega \right)}{P_{0}\left(\omega \right)}=\alpha \left(\omega \right)$ should define the absorption spectrum of the virus.

The latter is less than one for two reasons: (i) *scattering* and (ii) *absorption*. However, absorption strongly dominates because the Rayleigh scattering cross-section from a single nanoparticle (virus) is proportional to the sixth power of the diameter, while the absorption cross-section is proportional to the third power; see Appendix A.

The value of ω corresponding to the maximum of $\alpha \left(\omega \right)$ estimates the resonance frequency $\omega_{0}$, while the ratio between $\omega_{0}$ and the Full-Width at Half-Maximum (FWHM) of $\alpha \left(\omega \right)$, i.e., $\frac{\omega_{0}}{FWHM}$, estimates the quality factor Q.

In [22], the charge q was estimated by equaling, at the resonance frequency $\omega_{0}$, the theoretical absorption section (see Appendix B):

(5) $$\sigma_{abs}^{theor}\left(\omega_{0}\right)=\frac{q^{2}Q}{m\omega_{0}\varepsilon_{0}\sqrt{\varepsilon_{r}}c_{0}}$$

with the one given by the measurements (see Equation (13) in [22]). In Equation (5), m is the mass of the virus, $\varepsilon_{0}$ is the dielectric permittivity, $\varepsilon_{r}$ is the relative permittivity of the medium and $c_{0}$ is the speed of light.

The use of Equations (4) and (5) herein does not imply the acceptance of the underlying approach shown in [22], with its unjustified approximations; the only aim is to produce a rough order of magnitude evaluation of the entity of interaction between the e.m. field and the virus, as shown in the final part of Appendix A.

In [22], for the H3N2 virus, a reduced mass of core and shell, i.e., m=14.5 MDa ($2.41\cdot 10^{-17}\;g$) is used with respect to the total mass of 161 MDa ($2.67\cdot 10^{-16}\;g$); with $\varepsilon_{r}=67.13$, the experimental data of the microwave absorption spectrum provide $\omega_{0}=2\pi \cdot 8.2\;GHz$, Q=1.95 and a measured absorption section of $2.5\cdot 10^{-13}\;m^{2}$. Inverting Equation (5), the resulting value of the charge is as large as $q=1.16\cdot 10^{7}\;e^{-}$. Hence, for the H3N2 virus (average diameter of 100 nm) from [89], this results in $P_{stress}=0.141\;MPa$, and for the threshold of the electric field, Equation (4), $E_{0}^{T}=86.9\;V/m$.

This result obtained in [22] deserves some further exploration.

In [90], it is underlined that the density of charge is a distinguishing characteristic of each virus, depending crucially on the nature of the viral capsid and the presence/absence of the genetic material. In [91] we read, “*For a typical capsid surface with a net surface charge density σ of 0.1–1 charges per nm^2^, the extrapolation length is of order 0.1 nm*”. For a capsid with 100 nm diameter, the surface is about $3.14\times 10^{4}$ (nm^2^), for σ = 0.1 corresponding to 3140 charges, and 31,400 as a maximum. Hence, 3000 e^−^ to 30,000 e^−^ is a reasonable estimated range for the charge.

The value of the estimated charge $q=1.16\cdot 10^{7}\;e^{-}$ in [22] was not confirmed in any ensuing paper, to the best of our knowledge. We do agree that the parameter “*q*” is inherently difficult to estimate and may not be uniquely defined across the entire virion population. However, the common values of q reported in the literature are of the order of $10^{3}-10^{4}\;e^{-}$. For SARS-CoV-2, ref. [69] indicates the value $q=4650\;e^{-}$. Applying Equation (4) with a lower, more realistic charge q, the electric field threshold is high, greater than that of the dielectric breakdowns in air, approximately equal to 3 MV/m.

This result is confirmed in [58], where an atomic-scale Molecular Dynamics model was developed for the SARS-CoV-2 viral surface to estimate the electric field necessary to rupture the viral membrane via dipole shaking of the virus. The absorption spectrum was found to be essentially flat, i.e., no strong absorption was found in the GHz band (see also Appendix B.2). The investigation of the mechanical resiliency of the viral membrane by introducing uniaxial strains in the system showed no pore formation (no puncturing) in the membrane for strains up to 50%.

Such a situation requires $\frac{x_{max}}{r}>0.5$, where r is the radius of the virion and $x_{max}$ the maximum displacement relative to the center of the virion at $\omega =\omega_{0}$, i.e., from Equation (2):

(6) $$x_{max}=qE_{0}A\left(\omega_{0}\right)=\frac{qE_{0}Q}{m\omega_{0}^{2}}$$

In the supplementary information of [58], a 1−20 GHz band was considered with the following parameters for the virion: radius r=42 nm, $q∼10^{4}\;e^{-}$ (total charge on the virion), m∼203 MDa ($3.37\cdot 10^{-19}\;kg$) and Q=1. Choosing the lowest frequency, i.e., 1 GHz, and $x_{max}=\frac{1}{2}r$, the field-intensity $E_{0}$ for the rupture was minimized. From these data, by Equation (4), this results in $E_{0}=1.5\cdot 10^{8}\;V/m$, which is an order of magnitude less than the one estimated in [58] but still above the dielectric breakdown in air (3 MV/m).

These findings further support the hypothesis that dipole shaking is not a viable mechanism for the inactivation of viruses.

In fact, the authors of [58] concluded that *RF disinfection of enveloped viruses would occur only once sufficient heat was transferred to the virus via a thermal mechanism and not by direct action (shaking) of the RF field oscillations on the viral membrane.*

### 5.3. Patents on the Inactivation of Respiratory Viruses by Non-Thermal Microwave Devices

The great social and financial interest in the inactivation of viruses has justified important patent activity, particularly after the outbreak of COVID-19. A search on Espacenet—https://worldwide.espacenet.com/advancedSearch?locale=en_EP (3 September 2026) showed 546 results in the worldwide database for the words *virus–inactivation* in the title AND in the title or abstract, with only three results found in the same database for the words *microwave–virus–inactivation* in the title AND in the title or abstract.

The subset of patents related to the use of human-safe microwaves shows a very small number of patents; the interesting ones (according to our judgement) are listed as follows:

(a) U.S. patent No. 6,268,200 *“Biotherapeutic Virus Attenuation Using Variable Frequency Microwave Energy”*, Publication/Grant Date 31 July 2001 and Expiration Date 15 January 2019 (normal expiration after twenty years). IPC Classification A61L 2/00 (Sterilization/disinfection of special particles). The patent is for viral inactivation (attenuation) of lyophilized biotherapeutic products—such as blood-derived proteins or biologics—using variable frequency microwave (VFM) energy, selectively coupling water molecules inside the capsid—a thermally targeted mechanism. Assignee: Baxter International, Inc. and Lambda Technologies, Inc.
(b) U.S. patent Application No. 2001/0070624A1, 24 March 2011, *“Microwave resonant Absorption Method and Device for Viruses Inactivation”*. In the Summary we read, *“… a non-thermal method of inactivating the virus through microwave resonant absorption”*. Assignee: National Taiwan University, Taipei City, TW (appl. no. 12/562,591), hosting the research groups of [20,21,22]. This patent *is not active anymore*, as it was abandoned.
(c) EP4181966A1 *“Microwave disinfection system and method”* [40], an application filed by the Company Elettronica SpA on 24 May 2023, granted on 26 June 2024. From this patent, we quote, *“The sanitisation rate becomes much higher … when treatment is carried out in an aerosol. In this regard, the Applicant also carried out a second set of experimental aerosol inactivation tests of a solution containing SARS-CoV-2 using the following test parameters: 8–10 GHz band; 10 MHz steps; 3.2 s dedicated to each individual frequency for a total of 12 min of treatment; incident signal with a field amplitude of 100 V/m. … The above aerosol tests showed an inactivation percentage of SARS-CoV-2 equal to 83%”*.

## 6. Comments and Conclusions

For a field intensity of 6−15 V/m and an exposure of 2 min, the computation of the absorption cross-section in Appendix A shows that the transferred energy to a neutral droplet of water is too weak to generate any significant effect: it increases its temperature only by negligible amounts, ranging from $10^{-6}\;K$ to $10^{-4}\;K$ (see Table A4).

Although a considerable amount of the scientific literature has been devoted to the mathematical modeling of the SRET mechanism (see [20,59,67,92]), in our opinion the SRET mechanism was never demonstrated in viruses or in other organic material. In [93], we read, “*Although compact, morphologically well defined, viral nanoparticles have complex inner structures. Heterogeneous elasticity and/or diffuse structures of the several constituents (genome core, lipid layer,* etc.*) can prevent the development of motional coherence*”, and in the Conclusions, “*However, when it comes to the vibrational behavior, the hydrated viruses, in spite of the favorable ingredients like high morphological definition, cease to behave as solid nanospheres because they are unable to confine vibrations, unlike the polymer colloid counterparts*”, confirming that SRET/CAV is unlikely to exist in viruses.

Regarding [59], we observe the following:

(i) It is rather obvious that, assuming an electrical dipole model for the virus, it will interact with e.m. waves, whose frequency is related to the dipole length. The commonly used approximate solution (Equation (6) of [59]) for the resonance frequencies f for a dipole with length L (e.g., a rod-shaped virus) is, trivially, $f=\frac{nV}{\left(2L\right)},$ with *n* = 1, 2, …, and V as the longitudinal sound velocity. This oversimplified model, however, is not assessed by independent means.

(ii) The experiments are performed with viruses in solutions; they do not qualify inactivation means for respiratory viruses in the atmosphere.

The alleged transformation of a microwave photon into a phonon at the same frequency reduces its wavelength (in water, assuming a velocity of 1500 m/s) of a figure close to $2\cdot 10^{5}$. An 8 GHz microwave photon, becoming a phonon within the droplet, should change its wavelength from 3.75 cm to about 0.2 µm. This assumption is the only way to arrive in the resonance region for a significant part of the droplets or of the viruses. In fact, in [40] the Table on page 10, shown in the “Description of preferred embodiments of the invention”, expresses the resonance condition by the formula $f=\frac{2400}{2D}$, with f (in GHz) and where D (in nm) is the size of the virus.

To the best of our knowledge, a transformation, in water or in organic media, of a microwave photon into a phonon in the GHz interval has never been directly demonstrated and can hardly happen. Instead, microwave energy absorption in these media leads to heating, which generates many thermal phonons across a wide frequency spectrum, not a single coherent phonon at a specific frequency. Conversion of a single microwave photon to a single GHz phonon is a frontier of quantum research experimentally conducted in engineered systems (see for instance [94,95]), not in bulk material such as organic media. The transformation of microwave photons into phonons (mechanical or lattice vibrations) in organic media could occur through the coupling of electromagnetic fields to molecular degrees of freedom. However, this is hard to obtain due to the high intrinsic mechanical loss and to the thermal inhomogeneity generating local overheating under intense microwave fields, disrupting quantum phase coherence.

The above considerations are not intended to negate the relevance of the analysis of the acoustic response of viruses and bacteria, which encode information about structure and mechanical and biological states; see for instance [96].

Unclear points are found in [74], where a single pair of charges (equivalent to a single mass in the spring-mass model) is assumed, ignoring the more complicated structure of a virion (Figure 5); moreover, some confusion between absorption and scattering is present, such as in the sentence “*The effective absorption cross-section is therefore* $\sigma_{total}=\sigma_{abs}+\frac{\sigma_{s}}{2}$ ”, thus leading to wrong computations of the cross-section and of the viral titer.

The relationship between the percentage inactivation of viruses and the risk reduction (which obviously is not 1:1 nor linear) is analyzed in [26] and in the Airborne Risk Indoor Assessment (ARIA) model, developed and published by the World Health Organization (WHO). The ARIA framework provides a rigorous methodology to link reductions in viral loads to corresponding decreases in infection probability [97].

Finally, we read in [98]: *“The most recent development in disinfection systems is radiation-based systems. This category includes microwave, infrared (IR), and UV-C systems. Microwave-based and IR-based systems typically operate in an indirect manner. Namely, the microwaves (or radio-frequency waves) excite water, which thermally heats and destroys the spike glycoprotein. Thus, microwave radiation can be considered a moist thermal disinfection. Similarly, since IR sources are thermal sources, IR-systems can act as either dry or moist thermal systems”*. No mention is made of any non-thermal-microwave effect on the virions.

Summing up, we believe that the proposed inactivation of respiratory viruses by the alleged phenomenon of SRET, i.e., by means of radiated *human-safe* microwaves, has no significant effects on infecting droplets nor on viruses within the droplets.

The “virus inactivation device” by e4life (https://www.e4.life/it/prodotti/e4life-ambient/, accessed on 4 June 2026) complies with general European regulations regarding electromagnetic compatibility and electronic device safety, such as Directive 2014/53/EU (RED). We did not find any certification by a national or international health service/department, such as the ISS (Istituto Superiore di Sanità), concerning its anti-virus/disinfection effectiveness.

The fixed version of this device, designed to cover a 25 m^2^ room (say, a 5 m × 5 m room), is here assumed to emit a field within the 6 V/m limitation at short distances, say 1 m from the device’s horizontally pointing antenna. The field will decay (in the free space approximation) to 1.2 V/m at the opposite wall. Such a value is orders of magnitude below those referenced in the virus-inactivation literature.

Any kind of inactivation of respiratory viruses by e.m. radiation, in particular by microwave, depends on the basic parameters: intensity (W/m^2^), frequency, and exposure time. References [49,50,55,63,64,65,66] pay most attention to frequency and time and seem to neglect intensity. Moreover, their alleged validity of the SRET/CAV model is not confirmed by their inactivation results (see, for example, Figure 4 of [50] for the influenza virus), which appear to be wideband, not confirming the “photon-to-phonon” transformation and the resonant effects. Likely, the experienced, limited inactivation is related to e.m. intensity peaks in the analyzed droplet/aerosol volume.

## Figures and Tables

**Table 1:** Some relevant virus parameters of Influenza A and SARS-CoV-2.

Virus	Structure	Diameter	Mass
Influenza A [30]	Spherical (or pleomorphic) with a range of 109±13 nm in diameter	~100 nm	$\left(0.80\pm 0.35\right)\cdot 10^{-18}\;kg$
SARS-CoV-2 [31]	Spherical (or pleomorphic) with spikes on the surface with a length of 9–12 nm	~100 nm	$1.6\cdot 10^{-18}\;kg$

**Figure A1:**
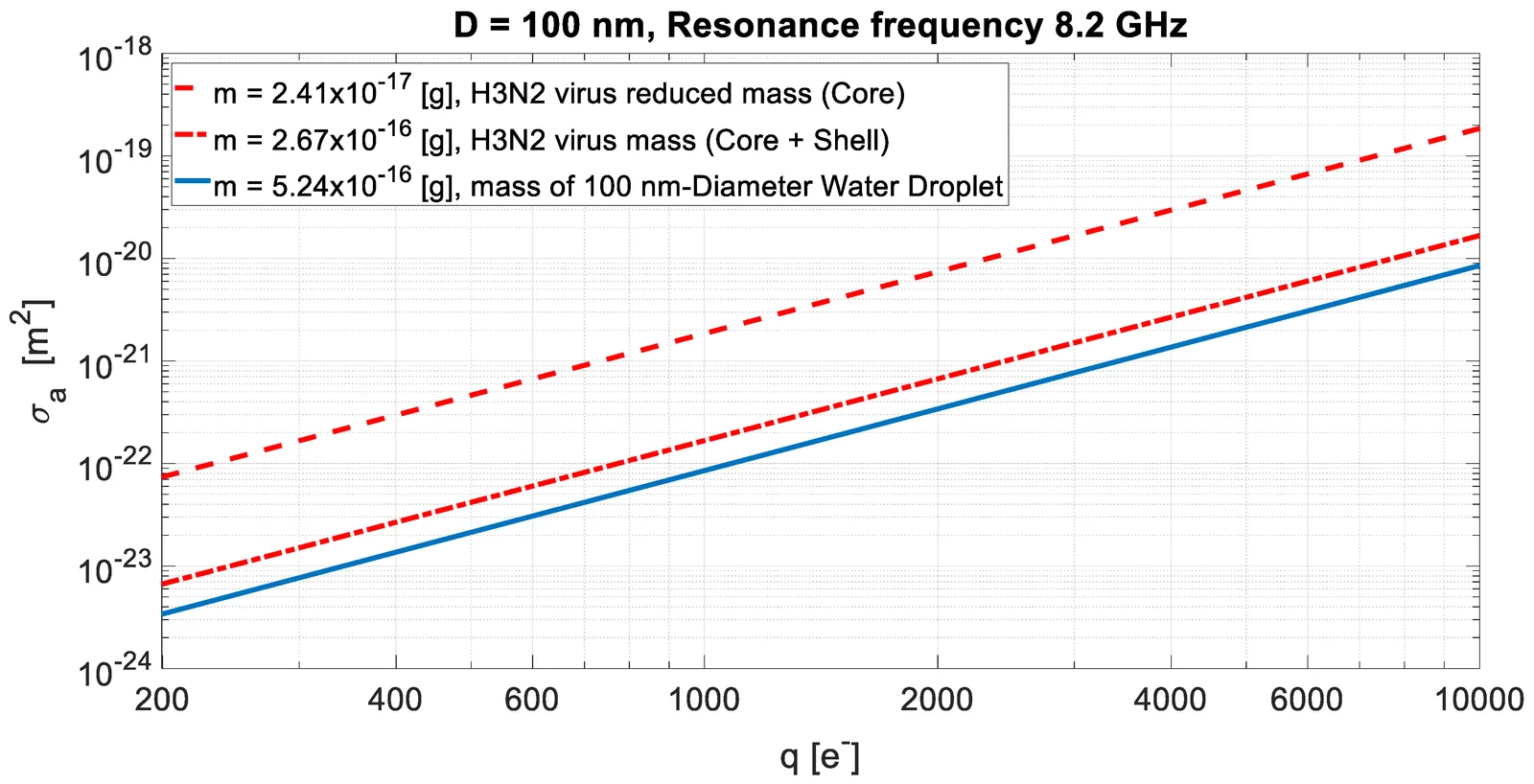
Absorption cross-section [$m^{2}$] versus the virus charge in electrons [$e^{-}$]. These values have to be compared with Equation (13) in [22].

**Figure A2:**
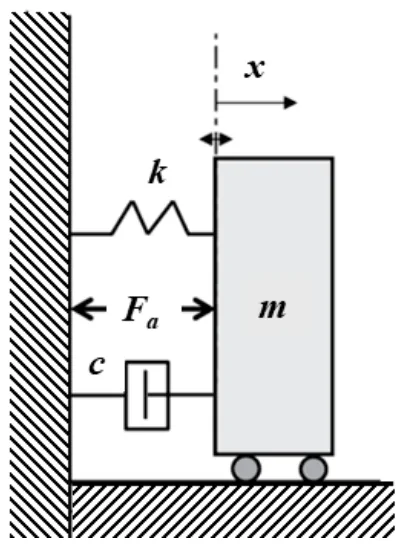
A damped mass-spring system.

**Figure A3:**
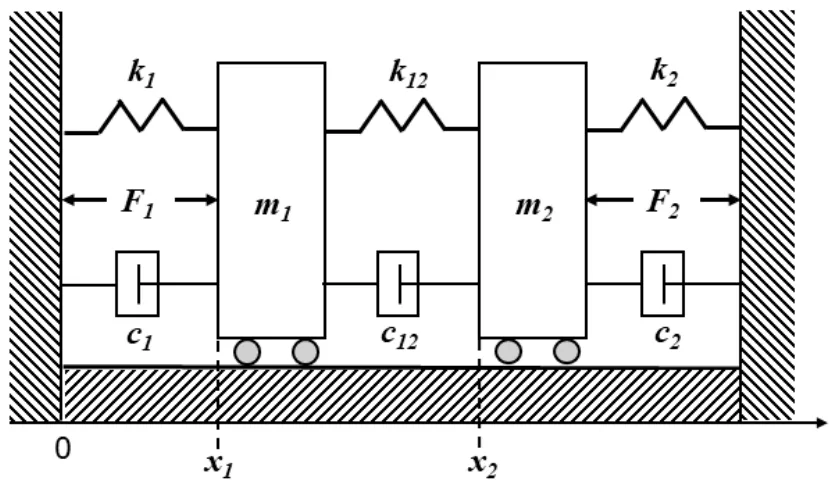
A double damped mass-spring system.

## Data Availability

No new data were created or analyzed in this study. Data sharing is not applicable to this article.

## Abbreviations

The following abbreviations are used in this manuscript:

CAV	Confined Acoustic Vibration
CPW	Co-Planar Waveguide
ERROR	Estimating the Reliability & Robustness Of Research
IEEE	Institute of Electrical and Electronics Engineers
ISS	Istituto Superiore di Sanità
MD	Molecular Dynamics
MRA	Microwave Resonant Absorption
SARS-CoV-2	Severe Acute Respiratory Syndrome CoronaVirus 2
SRET	Structure Resonance Energy Transfer
UV-C1	Ultra-Violet radiation, C1 band

## Appendix A. A Recall on e.m. Phenomena Related to Microwave Radiation on Water Droplets

As electromagnetic radiation propagates through the atmosphere, it interacts with particles, which in the present context are mainly droplets of water (diameter, D). These interactions result primarily in the form of *scatter* and *absorption*.

When a target is illuminated by an incident wave having a power density $S_{i}$, it will scatter/absorb a portion of the wave. The degree to which a “target” can scatter or absorb electromagnetic radiation is described through its cross-section σ, an equivalent area used to describe to what extent the radiation interacts with the target.

There are three different types of cross-section: the scattering cross-section $\sigma_{s}$, the absorption cross-section $\sigma_{a}$ and the extinction cross-section $\sigma_{e}=\sigma_{a}+\;\sigma_{s}$ (in radar applications, a fourth type is considered, i.e., the backscattering cross-section or *radar cross-section*).

The cross-section multiplied by the power density $S_{i}$ of the incident wave is equal to the total amount of power P removed from the electromagnetic wave through this process. In formula, it is represented as $P_{x}=S_{i}\cdot \sigma_{x}$, with x=a (absorption) or s (scattering) or e (extinction).

The German physicist Gustav Mie formulated (1908) a complete scattering/absorption theory [99], which describes the interaction of electromagnetic waves with spherical dielectric particles. When the droplet radius $a=\frac{D}{2}$ and the wavelength λ of the e.m. radiation satisfy the condition $\alpha =\frac{2\pi a}{\lambda }=\frac{\pi D}{\lambda }\ll 1$ (i.e., particles with a small radio-electric size), the Mie formulas are well approximated by the much simpler Rayleigh ones:

(A1) $$\sigma_{s}=\frac{2\;}{3}\frac{\pi^{5}}{\lambda^{4}}|{K_{m}|}^{2}D^{6}$$

(A2) $$\sigma_{a}=\frac{\pi^{2}}{\lambda }Im\left(-K_{m}\right)D^{3}\;$$

with $K_{m}=\frac{m^{2}-1}{m^{2}+2}$ and m=n−iκ being the complex refractive index of water droplets. The parameter m is related to the relative dielectric constant $\varepsilon_{r}=\epsilon^{\prime }+i\epsilon^{\prime \prime }$ by $\varepsilon_{r}=m^{2}$ and depends on the wavelength and on the temperature.

A dimensionless parameter is the Mie efficiency of absorption $e_{a}$ (resp., scattering $e_{s}$), equal to the ratio between the absorption/scattering cross-section and the physical cross-section, i.e., $\frac{{\pi D}^{2}}{4}$.

(A3) $$e_{s}=\frac{8}{3}\frac{\pi^{4}}{\lambda^{4}}D^{4}\;$$

(A4) $$e_{a}=\frac{4\pi }{\lambda }Im\left(-K_{m}\right)D\;$$

Note that from Equations (A1) and (A2), the ratio $\sigma_{s}/\sigma_{a}$ is proportional to ${\left(\frac{D}{\lambda }\right)}^{3}$, making the scattering contribution negligible with respect to the absorption one when D≪λ.

### Evaluation of the Microwave Radiation on Water Droplets

In the microwave region, with wavelengths from λ=0.03 m (f=10 GHz) to λ=0.06 m (f=5 GHz), at the ordinary temperature of 20 °C (293 K), the values of m, $|{K_{m}|}^{2}$ and $Im\left(-K_{m}\right)$ for pure water are shown in Table A1 [100].

For D=0.1 μm, 1 μm and 100 μm, using Equation (A2) and Equation (A4), we obtain the values of $\sigma_{a}$ and $e_{a}$ shown in Table A2. For D=100 μm, the absorption cross-section is of the order of $10^{-12}\;m^{2}$, i.e., is still negligible with respect to the physical cross-section of about $7.85\cdot 10^{-9}\;m^{2}$. The data shown in Table A2 agree with [100].

**Table A1:** Complex refractive index of water droplets m, $|{K_{m}|}^{2}$ and $Im\left(-K_{m}\right)$.

T=293 K				
	λ=0.03 [m] f=10.0 [GHz]	λ=0.0375 [m] f=8.0 [GHz]	λ=0.05 [m] f=6.0 [GHz]	λ=0.06 [m] f=5.0 [GHz]
m=n−iκ	8.0537−i2.0368	8.3330−i1.7371	8.5812−i1.3754	8.6876−i1.1723
$|{K_{m}|}^{2}$	0.9267	0.9273	0.9277	0.9279
$Im\left(-K_{m}\right)$	0.0196	0.0157	0.0118	0.0098

**Table A2:** Absorption cross-section and efficiency of water droplets.

	$\sigma_{a}\left[m^{2}\right]$			$e_{a}$		
$\lambda \left[m\right]$	D=0.1 µm	D=1 µm	D=100 µm	D=0.1 µm	D=1 µm	D=100 µm
0.03	$6.46\cdot 10^{-21}$	$6.46\cdot 10^{-18}$	$6.46\cdot 10^{-12}$	$8.23\cdot 10^{-7}$	$8.23\cdot 10^{-6}$	$8.23\cdot 10^{-4}$
0.0375	$4.14\cdot 10^{-21}$	$4.14\cdot 10^{-18}$	$4.14\cdot 10^{-12}$	$5.27\cdot 10^{-7}$	$5.27\cdot 10^{-6}$	$5.27\cdot 10^{-4}$
0.05	$2.33\cdot 10^{-21}$	$2.33\cdot 10^{-18}$	$2.33\cdot 10^{-12}$	$2.97\cdot 10^{-7}$	$2.97\cdot 10^{-6}$	$2.97\cdot 10^{-4}$
0.06	$1.62\cdot 10^{-21}$	$1.62\cdot 10^{-18}$	$1.62\cdot 10^{-12}$	$2.06\cdot 10^{-7}$	$2.06\cdot 10^{-6}$	$2.06\cdot 10^{-4}$

Multiplying $\sigma_{a}$ by the incident power density $S_{i}$, we obtain the absorbed power $P_{a}$. Considering an illumination (or exposure) time $t_{e}$, we obtain the absorbed energy $E_{a}=P_{a}$ $t_{e}$.

Table A3 shows the values of $P_{a}$ and $E_{a}$ for $t_{e}=120\;s$ with power density $S_{i}=9.55\cdot 10^{-2}\;W/m^{2}$, corresponding to $\sqrt{377\cdot S_{i}}=6\;V/m$, i.e., the upper permitted limit (at these frequencies) for radiation in the presence of persons (according to some national regulations, including Italy).

**Table A3:** Absorbed power $P_{a}$ [W] and absorbed energy $E_{a}$ [J].

$T=293\left[K\right],S_{i}=9.55\cdot 10^{-2}\left[\frac{W}{m^{2}}\right]=6\left[\frac{V}{m}\right]$						
$P_{a}[W]$				$E_{a}\left[J\right]$ with $t_{e}=120\left[s\right]$		
$\lambda \left[m\right]$	D=0.1 µm	D=1 µm	D=100 µm	D=0.1 µm	D=1 µm	D=100 µm
0.03	$6.17{\cdot 10}^{-22}$	$6.17\cdot 10^{-19}$	$6.17\cdot 10^{-13}$	$7.41\cdot 10^{-20}$	$7.41\cdot 10^{-17}$	$7.41\cdot 10^{-11}$
0.0375	$3.96\cdot 10^{-22}$	$3.96\cdot 10^{-19}$	$3.96\cdot 10^{-13}$	$4.75\cdot 10^{-20}$	$4.75\cdot 10^{-17}$	$4.75\cdot 10^{-11}$
0.05	$2.23\cdot 10^{-22}$	$2.23\cdot 10^{-19}$	$2.23\cdot 10^{-13}$	$2.67\cdot 10^{-20}$	$2.67\cdot 10^{-17}$	$2.67\cdot 10^{-11}$
0.06	$1.55\cdot 10^{-22}$	$1.55\cdot 10^{-19}$	$1.55\cdot 10^{-13}$	$1.86\cdot 10^{-20}$	$1.86\cdot 10^{-17}$	$1.86\cdot 10^{-11}$

Among the standard strategies to inactivate viruses, there is the use of UV-C radiation (λ=254 nm). At a wavelength between 250 nm and 350 nm, the complex refractive index of water at 20−25 °C shows the imaginary part of the order of $10^{-9}$ (see the website Refractive Index.info), i.e., pure water is largely transparent in the UV range, meaning that very little absorption occurs. UV-C radiation normally works with a power density of $50\;\mu W/cm^{2}$, corresponding to $0.5\;W/m^{2}(13.73\;V/m)$ which, compared with the RF density power of $424\;W/m^{2}$ (i.e., 400 V/m), results in significantly lower values, close to the human-safe limit.

Equation (A5) describes the increase of temperature ∆T (in Kelvin) when a droplet of water of mass $m_{D}=\frac{\pi }{6}D^{3}\rho_{w}$ ($\rho_{w}$ is the density equal to $1000\;kg/m^{3}$) is exposed to the e.m. wave for time $t_{e}$ (in seconds):

(A5) $$∆T=\frac{E_{a}}{\gamma \cdot m_{D}}=\frac{P_{a}\cdot t_{e}}{\gamma \cdot m_{D}}=6\frac{S_{i}\cdot \sigma_{a}\cdot t_{e}}{\gamma \cdot \pi D^{3}{\cdot \rho }_{w}}=6\cdot S_{i}\frac{\pi }{\lambda }\cdot \frac{Im\left(-K_{m}\right)\cdot t_{e}}{\gamma \cdot \rho_{w}}$$

where $\gamma =4184\;J\cdot kg^{-1}K^{-1}$ is the specific heat of water.

From Equation (A2), the absorption cross-section $\sigma_{a}$ depends on the third power of D; hence, ∆T is independent of D, and depends on the irradiated power density $S_{i}$, the exposure time $\left(t_{e}\right)$, the wavelength (λ) and the parameter $K_{m}$ of water, which depends on the temperature.

Considering three power density values, i.e., 6 V/m, 15 V/m ($S_{i}=0.5968\;W/m^{2}$) and 413 V/m ($S_{i}=452.4\;W/m^{2}$), and an exposure time of 120 s, we obtain for ∆T the values shown in Table A4. It is to be noticed that only the highest, non-personnel-safe intensity of 413 V/m shows a small, possibly biologically significant increase of temperature for the shortest wavelengths.

**Table A4:** The increase in temperature $∆T\;\left[K\right]$, shown in Equation (A5), when the droplet is exposed for a time of 120 s.

$∆T\;\left[K\right]\;\mathrm{at}\;t_{e}=120\;s$			
λ [m]	$S_{i}=9.55\cdot 10^{-2}\;[W/m^{2}]$ 6 [V/m]	$S_{i}=0.5968\;[W/m^{2}]$ 15 [V/m]	$S_{i}=452.4\;[W/m^{2}]$ 413 [V/m]
0.03	$3.38\cdot 10^{-5}$	$2.11\cdot 10^{-4}$	0.1600
0.0375	$2.17\cdot 10^{-5}$	$1.35\cdot 10^{-4}$	0.1030
0.05	$1.22\cdot 10^{-5}$	$7.62\cdot 10^{-5}$	0.0580
0.06	$8.47\cdot 10^{-6}$	$5.29\cdot 10^{-5}$	0.0400

The above evaluations do not include the contribution of the dipole-induced absorption by a virus within the droplet.

As a rough indication, using Equation (5) with the parameters reported in [22] for H3N2 virus, D=100 nm, $f_{0}=8.2\;GHz,\omega_{0}=2\pi f_{0}$, Q=1.95, and $\varepsilon_{r}=67.13,$ in Figure A1 we compare the absorption cross-section by the dipole factor (red dashed line for the only core mass and red dashed-dotted line for the total virus mass) with the one (blue line) obtained using for mass m that of a water droplet of the same diameter, 100 nm.

For a droplet of water (D=100 nm), at 8 GHz, the absorption cross-section is $\sigma_{a}=4.14\cdot 10^{-21}\;m^{2}$ (see Table A2), while for a virus with $q=5000\;e^{-}$, $\sigma_{a}$ increases up to $4.63\cdot 10^{-20}\;m^{2}$, a value significantly lower than the one measured in [22], i.e., $2.5\cdot 10^{-13}\;m^{2}$; see Equation (13) of [22].

## Appendix B. The Damped Mass-Spring System Applied to Virions

### Appendix B.1. The Case of a Single Mass (Single Pair of Charge)

Figure A2 shows a damped mass-spring system, where m is the mass of the body, k denotes the stiffness of the spring and c is the damping coefficient. The movement x by an external stimulus (the force $F_{a}$ of an actuator) is by the combined compliance of the body, the spring and the damper.

According to Newton’s laws, the force balance between $F_{a}$ and the resulting reaction forces is

(A6) $$m\frac{d^{2}x\left(t\right)}{dt^{2}}+c\frac{dx\left(t\right)}{dt}+k\cdot x\left(t\right)=F_{a}\left(t\right)$$

Using the Laplace transform, the differential Equation (A6) becomes

(A7) $$m\cdot s^{2}X\left(s\right)+c\cdot s\cdot X(s)+k\cdot X\left(s\right)=F_{a}\left(s\right)$$

From Equation (A7), we derive the transfer function of the system, where s=σ+jω (with j as the imaginary unit):

(A8) $$H\left(s\right)=\frac{X\left(s\right)}{F_{a}\left(s\right)}=\frac{1}{m\cdot s^{2}+c\cdot s+k}=\frac{1}{k}\cdot \frac{1}{\frac{m}{k}s^{2}+\frac{c}{k}s+1}\;$$

To qualify Equation (A8), three entities are introduced:

(A9a) $$the\;spring\;compliance:\;C_{s}=\frac{1}{k}$$

(A9b) $$the\;undamped\;natural\;frequency:\;\omega_{0}=\sqrt{\frac{k}{m}}\;\;\;$$

(A9c) $$the\;damping\;ratio:\;\zeta =\frac{c}{2\sqrt{k\cdot m}}=\frac{c}{2m\omega_{0}}$$

A fourth equivalent entity is

(A9d) $$the\;quality\;factor:\;Q=\frac{1}{2\zeta }$$

Hence, Equation (A8) becomes

(A10) $$H\left(s\right)=\frac{C_{s}}{\frac{s^{2}}{\omega_{0}^{2}}+2\zeta \frac{s}{\omega_{0}}+1}$$

To evaluate the permanent frequency response, we pose s=jω in Equation (A10):

(A11) $$H\left(\omega \right)=\frac{C_{s}}{1-\frac{\omega^{2}}{\omega_{0}^{2}}+j2\zeta \frac{\omega }{\omega_{0}}}$$

The magnitude of $H\left(\omega \right)$ is

(A12) $$A\left(\omega \right)=\left|H\left(\omega \right)\right|=\frac{C_{s}}{\sqrt{{\left(1-\frac{\omega^{2}}{\omega_{0}^{2}}\right)}^{2}+{\left(2\zeta \frac{\omega }{\omega_{0}}\right)}^{2}}}$$

Without damping (c=0, ζ=0), the response $A\left(\omega \right)$ becomes infinite at $\omega =\omega_{0}$, and the frequency $f_{0}=\frac{\omega_{0}}{2\pi }$ is the undamped *natural frequency* (or undamped *resonance frequency*) of the mass-spring system without damping. The system resonates when a periodic frequency of the stimulus force equals $f_{0}$.

In the presence of damping, ζ>0, at ${\omega =\omega }_{0}$ we have

(A13) $$A\left(\omega_{0}\right)=\frac{C_{s}}{2\zeta }=Q\cdot C_{s}=\frac{Q}{k}$$

where the quality factor Q of the damped mass-spring system quantifies the ability of a resonator to sustain its vibration over an extended duration of time.

From Equation (A11), the phase of $H\left(\omega \right)$ results in

(A14) $$\theta \left(\omega \right)=-arctan\left\{\frac{2\zeta \frac{\omega }{\omega_{0}}}{1-\frac{\omega^{2}}{\omega_{0}^{2}}}\right\}=-arctan\left\{\frac{\omega_{0}\omega }{Q\left(\omega_{0}^{2}-\omega^{2}\right)}\right\}$$

(A15) $$tan\left\{\theta \left(\omega \right)\right\}=-\frac{2\zeta \frac{\omega }{\omega_{0}}}{1-\frac{\omega^{2}}{\omega_{0}^{2}}}=-\frac{\omega_{0}\omega }{Q\left(\omega_{0}^{2}-\omega^{2}\right)}$$

At ω=0, the phase is zero, while at $\omega =\omega_{0}$ the phase is −90°. Increasing ω, the phase will be more negative, reaching −180° at ω=∞.

For real and sinusoidal stimulus, i.e., $F_{a}\left(t\right)=F\cdot cos(\omega t)$, the corresponding solution of Equation (A6) results in

(A16) $$x\left(t\right)=F\cdot A\left(\omega \right)cos\left\{\omega t+\theta \left(\omega \right)\right\}$$

Summing up, the permanent solution has the same frequency ω of the stimulus, with an amplitude related to $A\left(\omega \right)$ and a delay related to the phase $\theta \left(\omega \right)$.

The instantaneous power absorption can be evaluated by the product between the force F·cos(ωt) and the velocity of the mass along x: $v(t)=\frac{dx(t)}{dt}=F\cdot A\left(\omega \right)\cdot \omega \cdot sin\left\{\omega t+\theta \left(\omega \right)\right\}$.

Hence, the instantaneous power absorption is

(A17) $$P_{abs}(t)=F^{2}A\left(\omega \right)\omega \cdot cos(\omega t)sin\left\{\omega t+\theta \left(\omega \right)\right\}$$

and the average power absorption from the system is

(A18) $$\langle P_{abs}(t)\rangle =\frac{1}{2Q}F^{2}mA^{2}\left(\omega \right)\omega_{0}\omega^{2}$$

At resonance frequency,

(A19) $$\langle P_{abs}(t)\rangle =\frac{1}{2Q}F^{2}mA^{2}\left(\omega_{0}\right)\omega_{0}^{3}$$

With $A\left(\omega_{0}\right)=\frac{Q}{k}$ (see Equation (A13)), we have

(A20) $${\langle P_{abs}(t)\rangle }_{\omega =\omega_{0}}=\frac{F^{2}Q}{2m\omega_{0}}\;\;$$

Modeling a virion as a spherical homogeneous nanoparticle whose resonance absorption frequencies are those predicted by the elastic continuum theory, with the harmonic stimulus generated from an electric field $E\left(t\right)=E_{0}cos(\omega t)$, $F_{a}\left(t\right)$ of Equation (A6) becomes $F_{a}\left(t\right)=q\cdot E(t)=qE_{0}cos(\omega t)$, where q is the charge, $E_{0}$ is the incident electric field strength and the product $qE_{0}=F$ equals the parameter F of Equation (A20). Hence, Equation (A20) becomes

(A21) $${\langle P_{abs}(t)\rangle }_{\omega =\omega_{0}}=\frac{q^{2}E_{0}^{2}Q}{2m\omega_{0}}\;$$

Introducing the power flux S due to the e.m. field $E_{0}$,

(A22) $$S=\frac{1}{2}\cdot \frac{E_{0}^{2}}{Z}$$

where $Z=\sqrt{\frac{\mu_{0}}{\varepsilon_{0}\varepsilon_{r}}}$ is the impedance of the (non-magnetic) medium, $\mu_{0}=4\pi \cdot 10^{-7}\;\frac{H}{m}$ and $\varepsilon_{0}≅8.854\cdot 10^{-12}\;\frac{C^{2}}{Nm^{2}}$ are the magnetic and the dielectric permittivity, $\varepsilon_{r}$ is the relative permittivity of the medium, and the ratio $\frac{{\langle P_{abs}(t)\rangle }_{\omega =\omega_{0}}}{S}$ defines the theoretical absorption section:

(A23) $$\sigma_{abs}^{theor}\left(\omega_{0}\right)=\frac{q^{2}Q}{m\omega_{0}}Z=\frac{q^{2}Q}{m\omega_{0}}\sqrt{\frac{\mu_{0}}{\varepsilon_{0}\varepsilon_{r}}}=\frac{q^{2}Q}{m\omega_{0}\varepsilon_{0}\sqrt{\varepsilon_{r}}}\sqrt{\mu_{0}\varepsilon_{0}}=\frac{q^{2}Q}{m\omega_{0}\varepsilon_{0}\sqrt{\varepsilon_{r}}c_{0}}$$

where $c_{0}=\frac{1}{\sqrt{\mu_{0}\varepsilon_{0}}}$ is the speed of light.

### Appendix B.2. The Case of Multiple Masses (Pairs of Charge)

A virion can be realistically represented by more than two opposite electric charges, corresponding, in the frame of this model, to Figure A3 in the case of two pairs of charges (a double damped mass-spring system). Even in the over-simplified case with $F_{2}=0$, the frequency response related to $m_{1}$ is obtained solving an equation system in place of the single expression Equation (A7).

For the system in Figure A3, with masses $m_{1},m_{2}$, mass matrix $M=\left[m_{1}\;0\;0\;m_{2}\;\right]$, an interconnecting spring $k_{12}$, and stiffness matrix $K=\left[k_{1}+k_{12}\;-k_{12}\;-k_{12}\;k_{2}+k_{12}\;\right]$, the natural frequencies are found by solving the generalized eigenvalue problem [101,102]:

(A24) $$det\left(K-\omega^{2}M\right)=0$$

In general, a system with N pairs has $\frac{N(N+1)}{2}$ sets of parameters. For N identical masses m connected in a line by N+1 identical springs with stiffness k, the resonance frequencies are given by solving Equation (A24) with M and K $\left(N\times N\right)$ matrices. This results in

(A25) $$\omega_{i}=2\sqrt{\frac{k}{m}}sin\left(\frac{i\pi }{2\left(N+1\right)}\right)\;i=1,\;2,\ldots ,N$$

The model of identical springs hardly applies (Figure 5) to virions: the number of poles for the transfer function equivalent to Equation (A11) increases quadratically with N.

An external field acts on all pairs, and the model has to include the charge-to-charge interactions. This consideration justifies the appearance of multiple resonant frequencies for N>1, as in Figure 3 of [44].
